# Study on the influencing factors and composing path of online healthcare community use: an empirical study based on fsQCA

**DOI:** 10.3389/fpsyg.2025.1619464

**Published:** 2025-10-03

**Authors:** Zhanyou Wang, Dongmei Han, Liang Ma, Feifei Hao

**Affiliations:** ^1^School of Labor Relations, Shandong Management University, Jinan, China; ^2^School of Information Engineering, Shandong Management University, Jinan, China; ^3^School of Management Science and Engineering, Shandong University of Finance and Economics, Jinan, China; ^4^College of Traditional Chinese Medicine, Shandong University of Traditional Chinese Medicine, Jinan, China

**Keywords:** online healthcare community use, composing configuration, fsQCA, perceived usefulness, platform trust

## Abstract

In recent years, online healthcare communities have been widely adopted in China. However, the influencing factors and configuration paths affecting their usage remain unclear. This study employs Fuzzy-set Qualitative Comparative Analysis (fsQCA) to empirically investigate the determinants and combinatorial pathways leading to the use of online healthcare communities. Based on a cross-sectional survey design, data were collected via an online questionnaire using a snowball sampling method, resulting in 287 valid responses from users with experience in online healthcare platforms. The study examines condition configurations from the perspectives of patients, doctors, and platforms, identifying six sufficient combinatorial paths that lead to usage. Among these, the combination of perceived usefulness × perceived ease of use × platform trust exhibits the highest raw coverage. The findings enhance understanding of the mechanisms driving online healthcare community adoption and offer practical insights for platform optimization, policy-making, and future research.

## 1 Introduction

The online healthcare community has emerged as a vital platform, playing a pivotal role in healthcare delivery and development (Chen et al., 2022). It enables seamless communication between patients and healthcare professionals, fostering a collaborative approach to treatment and diagnosis (Zhu et al., 2022). Online healthcare community can bridge geographical gaps, ensuring that even remote areas have access to quality healthcare resources (Luo et al., 2022). In China, the development of online healthcare communities is closely tied to national health informatization strategies, such as the “Internet + Healthcare” initiative launched in 2018, which has strongly promoted the integration of digital technology and medical services. This policy-driven growth has led to the proliferation of domestic online healthcare platforms, with representative ones including Ping An Good Doctor (focusing on comprehensive online consultation and health management), AliHealth (integrating pharmacy services, appointment registration, and online diagnosis), and (centered on connecting patients with tertiary hospital experts for professional medical advice) (Chen et al., 2024). These domestic platforms not only conform to China’s medical system characteristics (such as the hierarchical diagnosis and treatment system) but also cater to Chinese users’ preferences for integrated services like medical insurance settlement links and offline hospital collaboration.

According to 2025 China Internet Development Report, the number of online healthcare users in China has reached 300 million by June 2025, with over 90% of these users accessing services through the aforementioned domestic platforms. This demonstrates the widespread adoption and popularity of domestic online healthcare platforms among Chinese citizens, as well as the dominant position of local platforms in the country’s online healthcare market (Hwang et al., 2022). However, study on the influencing factors and composing path of online healthcare community use–especially for these context-specific domestic platforms–is still not clear. Understanding the influencing factors cannot only help identify the barriers and enablers to the adoption of China’s domestic online healthcare communities but also provide insights into the effectiveness of these platforms in improving healthcare outcomes under China’s unique medical environment (e.g., alleviating the pressure of “difficult and expensive medical treatment” in large cities). Thus, it is important to study the influencing factors and composing path of online healthcare community use, with a focus on the domestic platforms that dominate China’s market.

A review of previous literature shows that many scholars have explored the factors influencing the use of online healthcare communities. Existing research has mainly focused on doctor factors, such as doctor trust, doctor professional quality, doctor skills, and so on (Guo et al., 2018; Li et al., 2020; Yang et al., 2021). However, these studies only analyzed single doctor attributes in isolation and did not explore how doctor factors interact with other dimensions. Platform factors, such as platform convenience, platform information resources, platform design, platform visibility, platform trust, platform information security, and so on (Ghose et al., 2022; Liu et al., 2020; Sun et al., 2022; Zhang et al., 2019). These studies, though rich in perspective, scattered platform factors into independent variables and failed to form a unified analytical framework for platform characteristics. Hospital factors, such as hospital grade, medical resources, hospital facilities, and so on (Yang J. X. et al., 2022; Zhou et al., 2022). These researches only described the existence of these factors and did not verify their combined effects on usage behavior. Medical environment factors, such as the impact of major emergencies, medical regulations and policies (Gopinathan et al., 2025; Zhang and Li, 2024; Zhu et al., 2022), most of these studies took macro factors as background variables rather than core antecedent conditions to analyze their configuration relationships with micro factors. It is also worth to pointed out that based on the Grounded theory, Wang et al. (2022) sorted out the influencing factors of the use of communities and the mechanism of the doctor-patient relationship from the perspectives of patients, doctors, online healthcare platforms, hospitals, and medical environments, and found patient factors, such as disease risk degree and patient evaluation. Existing research has made preliminary contributions to understanding the influencing factors of online healthcare community use, but there are two obvious gaps that this study aims to fill: First, lack of systematic integration of multi-dimensional factors. Most existing studies adopt a single dimension + single factor analytical logic (e.g., only discussing platform trust or doctor professional quality independently), and cannot explain the complex interaction between factors (e.g., whether platform trust can compensate for insufficient doctor trust to promote usage). Second, insufficient attention to the combination path of factors. Traditional quantitative studies often use regression models to test the independent effects of variables, ignoring the equifinality of usage behavior (i.e., different factor combinations may lead to the same usage outcome). Unlike these studies, this paper adopts the fsQCA method to integrate patient, doctor, and platform factors into a unified analytical framework, focusing on exploring the combinatorial configuration paths of multiple factors that drive online healthcare community use, and further clarifying the core conditional combinations that play a leading role in different scenarios.

The reasons are that: First, identify key causative pathways that influence usage behavior in the online healthcare community. Most of the existing results on the use of influencing factors in online healthcare communities are from the single variable thinking logic, and there is no systematic integration of influencing factors. The qualitative comparative analysis method can explore the relationship between the combination of multiple factors and the use behavior of the online healthcare community, identify the key causative path of the formation of the use behavior of the online healthcare community, and further determine the key intervention path of the use behavior management of the online healthcare community, laying a foundation for the exploration of the influential factors of the use behavior of the online healthcare community. Second, compared to a variety of causes of affecting the behavior of online healthcare community use path, and can be used for online healthcare community in different situation behavior to provide theoretical basis. Qualitative comparative analysis is different from traditional quantitative research in that it allows many different causative paths to exist. By analyzing the causative paths in different situations, corresponding guidance strategies can be formulated for the use behavior of online healthcare community, which will also provide multiple ways and possibilities for the intervention of the use behavior of online healthcare community. Third, fsQCA has been widely verified as a suitable method for analyzing complex consumer behavior and online platform usage in related research fields, and its advantages in this type of study are well-documented. For instance, Pappas et al. (2016) used fsQCA to explain online shopping behavior, emphasizing that it effectively captures the equifinality and complex interdependence of factors–characteristics that are also prominent in online healthcare community use (e.g., different combinations of patient, doctor, and platform factors may all lead to usage behavior). Similarly, Mikalef and Pateli (2017) applied fsQCA to study information technology-enabled dynamic capabilities in online platforms, demonstrating that the method can accurately identify non-linear relationships between multiple antecedent factors and platform-related outcomes. In the context of healthcare, Yu et al. (2025) also adopted fsQCA to explore configuration effects in healthcare-related behavioral research, further confirming that fsQCA is more capable of handling the complexity of healthcare user behavior compared to traditional linear regression methods that assume independent factor effects. Thus, using fsQCA aligns with the methodological trends in related fields and ensures the validity of analyzing the complex configuration paths of online healthcare community use.

This paper uses the qualitative comparative analysis method to explore the combination of key causes of the use behavior in online healthcare communities, and then explores the combination intervention path of the use behavior in online healthcare communities. Through the introduction of this method, this paper has made the following implications. Firstly, this paper contributes to the online healthcare community literature by providing a systematic and comprehensive understanding of the complex factors that influence the usage of online healthcare communities. Secondly, this paper contributes to the online healthcare community literature by the identification of key cause combinations and intervention paths offers valuable insights for optimizing and improving online healthcare community services. Finally, the findings of this paper have practical implications for the design and implementation of online healthcare community platforms.

## 2 Research model

In the qualitative study on the formation mechanism of use behavior in online healthcare communities, Wang et al. (2022) identified five dimensions of influencing factors, including patient-related, doctor-related, platform-related, medical environment related and medical treatment related, and built their formation mechanism model. In the actual exploration of influencing factors, we initially attempted to retain the “hospital-related” and “medical environment” variables for comprehensive analysis, as these factors have been proven to have potential impacts on healthcare service adoption in existing literature (Gopinathan et al., 2025; Yang J. X. et al., 2022). However, after in-depth feasibility verification and pre-survey tests, we ultimately decided to exclude these two types of variables, with the specific reasons clarified as follows:

First, regarding hospital-related variables (e.g., hospital grade, medical resources, hospital facilities), the core challenge lies in the difficulty of accurate measurement and the ambiguity of their direct association with online healthcare community use. In the context of China’s domestic online healthcare platforms (e.g., https://www.haodf.com/, Ping An Good Doctor), most platforms cooperate with multiple hospitals at different levels (from primary community hospitals to top tertiary hospitals), and users often access doctor resources through the platform rather than directly selecting services based on the hospital itself. This means that users’ perception of hospital factors is often mediated by the platform’s doctor resource integration, making it difficult to disentangle the independent impact of hospital-related variables from platform-related variables. For example, a user’s trust in a doctor on the platform may stem from the doctor’s professional background rather than the hospital they belong to. Additionally, hospital-related variables (such as hospital facility quality) are inherently offline attributes, and their correlation with the online behavior of using healthcare communities is weak–users cannot perceive or evaluate offline hospital facilities through online interactions, leading to low validity of measurement items.

Second, for medical environment variables (e.g., impact of major emergencies, medical regulations and policies), the key issue is the stability of factor effects and difficulty in capturing individual differences. On the one hand, medical environment factors are often macro-level and time-sensitive, could not reflect the long-term, stable influencing mechanism of online healthcare community use. On the other hand, medical regulations and policies are universal constraints for all users and platforms, and there is no significant individual difference in users’ perception of these policies–nearly all respondents in the pre-survey reported that they only know that online healthcare platforms are legal, but do not understand specific policy details, resulting in a lack of variability in measurement data, which would reduce the discriminatory power of the model.

**FIGURE 1:**
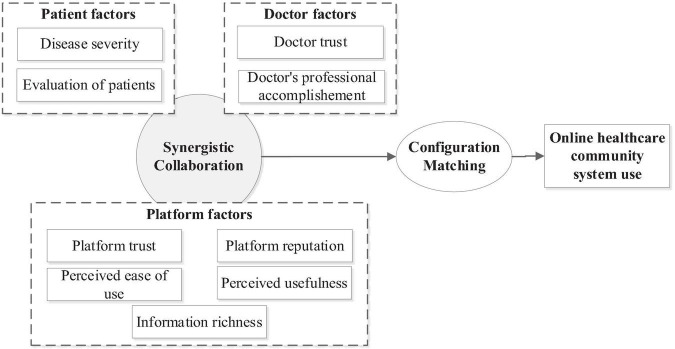
Model of influencing factors of use behavior in online healthcare community.

To ensure the reliability and validity of the research model, and to focus on the factors that have direct, measurable, and stable impacts on users’ online healthcare community use behavior, this paper removes the hospital-related and medical environment variables and only considers three categories: patient-related, doctor-related and platform-related. Patient-related factors included disease severity and patient evaluation. Platform-related factors include platform visibility, information richness, medical platform trust, perceived ease of use, perceived usefulness; Factors related to doctors include doctor trust and doctor professional accomplishment. The specific situation of the research theoretical model constructed in this paper according to TAM theory is shown in the Figure 1 below.

## 3 Materials and methods

### 3.1 Questionnaire design

This paper adopts the method of questionnaire survey to carry out relevant research. First of all, based on the scale of Wang et al. (2023), the scale is optimized through verification, and on this basis, the questionnaire of this paper is designed. The final measurement items of the scale are shown in Table 1. The questionnaire was presented on a 7-point Likert scale ranging from 1 (strongly disagree/unlikely) to 7 (strongly agree/very likely).

**TABLE 1:** Measurement items.

Variable	Measure items
Platform reputation (PR)	I know the online healthcare community very well
	Online healthcare communities have a reputation
	As far as I know, the online healthcare community is also promoted through various channels such as offline and online
Doctor’s trust (DT)	I think the doctors in the online healthcare community will put my life safety and health concerns first
	I think the diagnosis and treatment plan and health advice provided by doctors in online healthcare communities are scientific and reasonable
	I think the doctors in the online healthcare community are willing to communicate with me about my illness and share health knowledge
	I believe that the doctors in the online healthcare community will make efforts for my physical health and recovery from illness
	I think the online healthcare community doctor is really patient-centered
Information richness (IR)	Online healthcare communities have access to doctor information
	Online healthcare communities can access registration information
	Online healthcare communities can access consultation information
	Online healthcare communities have access to medication information
	Online healthcare communities are able to access medical experience
	Online healthcare communities have access to patient evaluation information
Platform trust (PT)	The online healthcare community platform has professional qualification certification, and the medical staffs on the platform have professional qualification certification and rich experience in diagnosis and treatment.
	I believe that the content of interactions with doctors and other users through online healthcare communities is private and safe.
	The online healthcare community provides me with medical advice, disease information and health ideas and other content is scientific.
	In my opinion, online healthcare communities can effectively alleviate the information asymmetry between doctors and patients.
	The online healthcare community provides me with reliable medical advice, disease information and health ideas
	I think the medical science articles published by the online healthcare community can increase my trust in the online healthcare community
Perceived ease of use (PE)	Learning to use the online healthcare community was easy for me
	It is easy to consult a doctor on the online healthcare community
	The operation interface of the online healthcare community is friendly and easy to understand
	It is not difficult to become proficient in using online healthcare communities
	The online healthcare community function is designed based on the patient’s perspective and is very user-friendly
Disease severity (DS)	When I used the online healthcare community, the illness was serious
	When I use the online healthcare community, the illness is urgent
	When I use online healthcare communities, the symptoms of illness are mixed
Patient evaluation (PCA)	Online healthcare community patient evaluation are referable
	Online healthcare community patient evaluations are real
	Online healthcare community patient reviews are credible
	Online healthcare community patient evaluation are important
	Online healthcare community evaluation are effective
Doctor’s professional accomplishment (DP)	Online healthcare community doctors have a better attitude
	Online healthcare community doctors have good medical ethics
	Online healthcare community doctors have certain skills
	Online healthcare community doctors have rich experience in diagnosis and treatment
	Online healthcare community doctors have professional spirit
Perceived usefulness (PU)	It is useful for online healthcare communities to provide services such as registered appointments and patient consultations
	The use of online healthcare communities can improve the efficiency of booking appointments
	Use online healthcare communities to save time
	Online healthcare communities have advantages over traditional offline registration
	Using online healthcare communities can reduce the cost of medical care (economic cost of medical care)
Intention to use (UI)	I think online healthcare community is a good way for doctors to communicate with patients
	I would like to use the online healthcare community in case of health problems
	With the spread of information technology, I think it is a popular trend to use online healthcare communities
	I would like to recommend friends to use the online healthcare community
	I am willing to use online healthcare communities on the recommendation of others

### 3.2 Data collection

After the questionnaire design was completed, we conducted an online questionnaire survey. There are various methods for questionnaire survey. This study chooses the “snowball” method for online survey, which has the characteristics of low cost, rapid, convenient and rapid, and can ensure the selection of a large sample of consumers and improve the effectiveness of questionnaire filling. In this study, the questionnaire was designed with the help of the questionnaire star developed by the leading Internet research company in China. Then, from February to March 2021, a “snowball” approach was adopted to conduct the online survey. Each participant was asked to have experience using the online healthcare community, and was asked to fill out a questionnaire to collect their personal information and personal experience using the online healthcare community platform. The questionnaire first briefly describes the purpose of the survey, namely to explore the online healthcare community (e.g., Then interviewees are required to simply fill in their basic personal information, including gender, age, education level, monthly income, health status and other information, and finally share their platform use and online healthcare treatment experience based on their own experience in using online healthcare community platform. After the official questionnaire was released, a total of 291 respondents participated in the online questionnaire survey. Among the recovered questionnaires, 287 were valid, with an effective rate of 98.63%.

This study was conducted in accordance with the ethical guidelines of the Declaration of Helsinki. All participants provided informed consent before completing the survey. They were informed about the research purpose, data usage, and their right to withdraw at any time. Anonymity and confidentiality were ensured throughout the data collection and analysis process. The study protocol was approved by the Ethics Committee of Shandong University of Management.

### 3.3 Data analysis tool

This paper uses Smart PLS3.0 to analyze the data and test the hypotheses. PLS3.0 is a second generation multivariate statistical data analysis tool that evaluates both measurement and structural models (Ma et al., 2020). PLS model has a small sample size and is not limited by normal distribution (Ma and Zhang, 2025; Ma et al., 2021, 2024), so it is more suitable for current research. Following a two-step analysis step, the measurement model and the structural model are tested, respectively.

## 4 Data analysis

### 4.1 Descriptive statistics

Table 2 lists the sample characteristics of the collected questionnaires. It can be seen that the respondents were mostly female, accounting for 60.98% of the questionnaire, while male respondents accounted for 39.02%. This gender distribution, with a higher proportion of females, is not random but aligns with the actual characteristics of online healthcare community usage in China and the specific context of this study’s data collection. The main reasons are as follows: First, from the perspective of user behavior characteristics in China’s online healthcare scenarios, existing research [e.g., Yang M. et al. (2022)] has consistently shown that females are more likely to engage in health management behaviors–such as actively seeking medical information, consulting about chronic diseases, or arranging health checks for family members–compared to males. This higher health engagement motivates females to use online healthcare communities more frequently, making them more willing to participate in surveys focused on platform usage experience.

**TABLE 2:** Descriptive statistics of interviewee characteristics.

Project	Attribute	Number	%
Gender	Male	112	39.02%
	Female	175	60.98%
Age	≤20 years old	8	2.79%
	21–30 years old	28	9.76%
	31–40 years old	43	14.98%
	41–50 years old	174	60.62%
	>50 years old	34	11.85%
Education	Senior middle school or below	146	58.15%
	Junior college	51	17.77%
	Bachelor’s degree	40	13.94%
	Master’s degree	32	11.15%
	Doctor’s degree or above	18	6.27%
Income	<3000	95	33.10%
	3000–4999	77	26.83%
	5000–7999	60	20.91%
	8000–11999	36	12.54%
	≥12000	19	6.62%
Physical condition	Very good	86	29.97%
	Relatively good	108	37.63%
	General	85	29.62%
	Relatively poor	8	2.79%

In terms of age, most respondents are between 41 and 50 years old, accounting for 60.62% of the questionnaire, followed by respondents between 31 and 40 years old, accounting for 14.98% of the questionnaire, and respondents over 50 years old accounted for 11.85%. Meanwhile, 9.76% of respondents were aged between 21 and 30, while those aged 20 and below accounted for 2.79%. In terms of education, most of the respondents are high school or below, accounting for 50.87% of the questionnaire, followed by 17.77% of the respondents are college degree, 13.94% of the respondents are bachelor degree, 11.15% of the respondents are master degree, and 6.27% of the respondents are doctor degree. In terms of monthly income, most of the respondents are less than 3,000 yuan, accounting for 33.10% of the respondents, followed by 26.83% whose income is between 3000 and 4999 yuan, 20.91% whose income is between 5000 and 7999 yuan, and 12.54% whose income is between 8000 and 11999 yuan. Respondents with more than 12,000 yuan accounted for 6.62%. Regarding the health status of the respondents, 37.63% of the respondents were in good health, 29.97% were in very good health, 29.62% were in average health, and 2.79% were in poor health.

The basic characteristics of the sample data are summarized and explained through descriptive statistical analysis to ensure that the relevant requirements of subsequent inferential statistics are met. SPSS software was used to process sample data and calculate the mean value, standard deviation, variance, skewness and kurtosis of sample data. According to the data shown in the table, the mean value of each item is relatively balanced, and the degree of dispersion is completely within the acceptable range. The absolute value of skewness of each item is between 0.14 and 1.8, and the absolute value of kurtosis of each item is between 0.00 and 5.45. When the sample size is greater than 200, the absolute value of skewness is less than 2 and the absolute value of kurtosis is less than 7. Therefore, it can be considered that the sample data in this study meet the requirements of normal distribution.

### 4.2 Measurement model

The measurement model can be evaluated by testing reliability, aggregation validity, and discriminative validity. Specifically, reliability can be assessed using Cronbach’s alpha, composite Reliability (CR), and average variance extracted (AVE). According to the suggestions of Ma et al. (2021), Cronbach’s alpha is accepted when it is greater than 0.7, CR is accepted when it is greater than 0.7, and AVE is accepted when it is greater than 0.5. Table 3 shows that Cronbach’s alpha ranges from 0.829 to 0.941, far exceeding the recommended value of 0.7. The CR range is 0.898 ∼ 0.962, far exceeding the recommended value of 0.70. The AVE ranges from 0.666 to 0.894, well above the recommended level of 0.5.

**TABLE 3:** Reliability and validity test.

Variable	Cronbach’s Alpha	rho_A	CR	AVE
DP	0.941	0.943	0.955	0.809
DT	0.927	0.928	0.945	0.773
DS	0.941	0.947	0.962	0.894
IR	0.900	0.902	0.923	0.666
PCA	0.919	0.92	0.939	0.756
PE	0.915	0.916	0.936	0.745
PR	0.829	0.836	0.898	0.746
PT	0.912	0.915	0.932	0.695
PU	0.887	0.888	0.917	0.689
UI	0.916	0.92	0.937	0.748

Through the above analysis, the measurement structure has a high reliability. Aggregation validity can be evaluated by factor loading. In this paper, all factor loads are higher than 0.7, indicating that the convergence validity is good. At the same time, the discriminative validity of the measurement items is also tested. As can be seen from Table 4, the AVE square root of each variable is greater than its correlation coefficient with other factors, indicating that the model has good discrimination validity.

**TABLE 4:** Descriptive statistics and internal structural correlations.

Variable	DP	DT	DS	IR	PCA	PE	PR	PT	PU	UI
DP	0.899									
DT	0.839	0.879								
DS	0.258	0.231	0.945							
IR	0.702	0.715	0.198	0.816						
PCA	0.741	0.777	0.38	0.71	0.87					
PE	0.563	0.615	0.126	0.571	0.595	0.863				
PR	0.561	0.539	0.382	0.556	0.572	0.57	0.864			
PT	0.741	0.772	0.313	0.831	0.773	0.595	0.655	0.834		
PU	0.545	0.621	0.094	0.617	0.595	0.755	0.577	0.612	0.83	
UI	0.56	0.606	0.165	0.571	0.602	0.732	0.527	0.641	0.699	0.865

DP, doctor’s professional accomplishment; DT, doctor trust; DS, disease severity; IR, information richness; PCA, patient evaluation; PE, perceived ease of use; PR, platform reputation; PT, platform trust; PU, perceived usefulness; UI, intention to use.

In order to ensure that the data set is not affected by common method bias, this paper adopts Harman’s single factor analysis method for testing. The measurement of all variables is analyzed by a single factor to further observe the extent to which this single factor explains all variables. The statistical results show that no single factor can be present, and that the first factor accounts for 36.606% of the variance, which is less than the recommended threshold of 50% (Podsakoff et al., 2003). As a result, the dataset does not have any problems with common methodology biases.

## 5 Results of fsQCA

The essence and basis of qualitative comparative analysis method is still case study, which is an optimization and improvement of solving “within case” and “cross-case” analysis method (Huarng and Roig-Tierno, 2016). Qualitative comparative analysis method has the following three characteristics: First, from the perspective of local characteristics, cases can be solved into multiple variables; from the perspective of overall characteristics, different variables interact with each other, and variable interaction and combination can represent the whole case to a certain extent. Secondly, the qualitative comparative analysis method focuses on the comparison and analysis between different cases, through which the integration and deepening of case information can be realized, and the influence of the combination effect of factors on the results can be analyzed. However, traditional research often studies a single case or cross-case, and cannot analyze the interaction between factors or the combination of factors. Qualitative comparative analysis is more closely related to practice and can help better solve problems in practice. Third, qualitative comparative analysis can evaluate the combination effect of multiple factors. Through comparative analysis of the combination of multiple factors that lead to the same result, different theories can be generated in different situations.

Before using the fsQCA method for data analysis, it is necessary to perform proportional calibration (Guo et al., 2022). The correction step consists of calculating the mean value of each structure, identifying three fuzzy conversion indicators as full membership (1), cross point (0.5), and full non-membership (0), and finally converting the raw data to continuous data from 0 to 1 by calculating scalar and logarithmic summation (Jolyane and Nabil, 2023). FsQCA solutions are evaluated based on two metrics: consistency and coverage. Sufficient consistency is a prerequisite for testing set coverage, and researchers recommend using a consistency threshold of at least 0.75–0.95 (Dul, 2016). Researchers can select a consistency threshold by looking at the number of distributions of consistency and selecting the score that corresponds to the gap (Xie and Wang, 2020). In the final solution, researchers should preserve all combinations of causes for which the display value is above the selected conformance threshold. In addition to consistency measures, coverage statistics are also used to interpret fsQCA findings (Mikalef and Pateli, 2017). Coverage measures the empirical importance of the solution to achieving the results of interest. Specifically, it shows the solution as a whole and how many outcomes are explained by each solution path individually. When coverage is between 0.25 and 0.65, the model solution is considered interpretive. The higher the consistency threshold selected, the lower the corresponding coverage (Pappas et al., 2016).

### 5.1 Variable calibration

In this study, the relevant data obtained by Likert 7-point scale were used to measure each variable with 3–5 items, and the average score of each variable was taken as the final score of the variable. Based on the research results of Kumar et al. (2022) and the actual situation of collected data, the average plus one standard deviation, the average plus one standard deviation and the average minus one standard deviation are used as calibration anchors to avoid contradictory structures.

### 5.2 Analysis of necessary conditions

In order to determine whether a certain condition is a necessary condition for the result, this study first conducted a necessity test for a single factor, and the test results are shown in Table 5. In general, a concordant level of more than 0.9 indicates that the condition is necessary to give rise to the result. According to the research results, the consistency value of all the antecedent variables in this study is less than 1, indicating that these antecedent variables are not necessary conditions for the outcome variables, indicating that the antecedent variables of influencing factors in the use of online healthcare communities are not necessary conditions for the use behavior of online healthcare communities, and further configuration analysis is needed.

**TABLE 5:** Antecedent condition necessity test.

Antecedent condition	UI		Antecedent condition	∼UI	
	Consistency	Degree of coverage		Consistency	Degree of coverage
PE	0.83	0.84	PE	0.43	0.35
∼PE	0.35	0.44	∼PE	0.80	0.79
PU	0.83	0.83	PU	0.46	0.37
∼PU	0.36	0.46	∼PU	0.79	0.79
PR	0.73	0.79	PR	0.42	0.36
∼PR	0.41	0.47	∼PR	0.76	0.69
IR	0.79	0.79	IR	0.48	0.38
∼IR	0.38	0.48	∼IR	0.74	0.74
PT	0.79	0.81	PT	0.43	0.35
∼PT	0.37	0.45	∼PT	0.77	0.74
DS	0.62	0.67	DS	0.55	0.47
∼DS	0.50	0.58	∼DS	0.61	0.56
PCA	0.78	0.81	PCA	0.42	0.34
∼PCA	0.36	0.44	∼PCA	0.77	0.74
DT	0.80	0.81	DT	0.46	0.37
∼DT	0.37	0.46	∼DT	0.76	0.75
DP	0.79	0.81	DP	0.42	0.35
∼DP	0.37	0.45	∼DP	0.77	0.74

### 5.3 Configuration analysis

In this study, fsQCA3.0 was used to analyze the case data. By selecting the frequency threshold as 1 and the consistency threshold as greater than 0.8, the combination of conditions with poor representativeness and universality was excluded, and the condition configuration that significantly caused the results was retained. Standard analyses are selected, and the output is three kinds of results: complex solution, parsimonious solution and intermediate solution. Among them, the intermediate solution is a result that is completely set according to variables in qualitative comparative analysis, and is also the preferred analysis scheme in fsQCA analysis. According to the coverage index, the explanatory power of configuration on the use of substance in online healthcare community is 0.708. Six configurations together explain the common path used by the online healthcare community. The 6 specific sufficient condition combination paths found in the study are shown in Table 6 for specific analysis results.

**TABLE 6:** Antecedent conditional configuration.

Conditional combination	Original coverage	Unique coverage	Consistency
pe × pu × pt	0.634417	0.026082	0.891789
pe × pu × pca	0.615662	0.029302	0.915141
pt × ∼ds × pca × dp	0.330218	0.030250	0.846390
pu × ∼pr × ir × pt × ∼pca	0.117146	0.016672	0.826281
pe × ∼ir × dp	0.108494	0.010862	0.886482
pu × ∼pr × pca × ∼dt	0.102305	0.001516	0.873787
Overall coverage:0.821030			
Global consistency:0.839587			

Path 1: Perceived usefulness × perceived ease of use × platform trust (original coverage: 0.634417; Consistency: 0.891789). This indicates that the platform usefulness, platform ease of use and platform trust constitute the most important path for users to use online healthcare community. This path reflects China’s integrated “Internet + Healthcare” policy, where platforms must partner with public hospitals and obtain official qualifications, building user trust through policy endorsement. Perceived usefulness is heightened by features like online healthcare community use, which address specific local needs and drive adoption.

Path 2: Perceived ease of use × perceived usefulness × patient evaluation (original coverage: 0.615662; Consistency: 0.915141). This indicates that the ease of use of online healthcare community platform, platform usefulness and patient evaluation are the important paths for users to use online healthcare community. This path highlights the role of patient evaluations in China’s healthcare culture, where users place strong trust in peer experiences–especially detailed, illness-specific reviews from those with similar conditions. Platforms like leverage this by featuring authentic consultation feedback, which significantly influences new users’ adoption decisions. This reliance on communal word-of-mouth is a distinctive feature of China’s online healthcare behavior.

Path 3: Platform trust × ∼ disease severity × patient evaluation × doctor professional quality (original coverage: 0.330218; Consistency: 0.846390). This indicates that online healthcare community platform trust, non-disease severity, patient evaluation and doctor professionalism constitute an important path for users to use online healthcare community. This path aligns with China’s regulatory framework, where online platforms are restricted from handling severe or acute cases and instead focus on minor illnesses and chronic disease management. Users thus turn to these communities for non-severe needs, driven by trust in the platform and the perceived professionalism of doctors. Positive patient evaluations further reinforce this by validating doctors’ effectiveness within these approved service boundaries.

Path 4: Perceived usefulness × ∼ perceived risk × information richness × platform trust × ∼ patient evaluation (original coverage: 0.117146; Consistency: 0.826281). This indicates that perceived usefulness, non-perceived risk, information richness, platform trust and non-patient evaluation constitute important paths for users to use online healthcare communities. The perceived risk and information richness factors reflect China’s strengthened regulation of online healthcare information, such as the 2023 National Health Commission campaign to combat false medical content. This policy has reduced users’ perceived risk and elevated the value of authoritative, platform-verified information–particularly among middle-aged and elderly users who prioritize accuracy. Consequently, trust in official sources has grown, sometimes surpassing reliance on peer evaluations.

Path 5: perceived ease of use × ∼ information richness × physician professional quality (original coverage: 0.108494; Consistency: 0.886482). This indicates that the perceived ease of use, non-information richness and doctor professionalism of online healthcare community constitute the important paths for users to use online healthcare community. This path reflects the needs of users in China, who prioritize ease of use–such as simplified interfaces and voice support–over information richness due to digital barriers. They also highly value doctors’ professional qualifications, like senior titles and public hospital affiliations, which platforms prominently display. A simple, trustworthy interface with credible doctors sufficiently drives adoption among this group.

Path 6: Perceived usefulness × ∼ perceived risk × patient evaluation × physician trust (original coverage: 0.102305; Consistency: 0.873787). This indicates that the perceived usefulness, non-perceived risk, patient evaluation and doctor trust of online healthcare community constitute the important paths for users to use online healthcare community. This path reflects the importance of institutional trust in China’s healthcare context, where users rely heavily on doctors’ affiliations with top public hospitals and their specialized reputations. Stringent platform regulations–such as mandatory record-keeping and dispute channels–reduce perceived risk. Trust is thus built through institutional credibility and procedural security rather than solely through online reviews.

### 5.4 Robustness test

To ensure the reliability and stability of the fsQCA configuration results, this study strictly followed the robustness test framework for qualitative comparative analysis and adopted two set theory-based methods proposed by Kumar et al. (2022)–a widely recognized approach in QCA research for verifying result consistency–to systematically validate the robustness of the identified 6 configuration paths.

As shown in Table 7, both robustness test methods confirmed that the 6 configuration paths driving users’ intention to use online healthcare communities–including the high-coverage core paths (Path 1: PE × PU × PT; Path 2: PE × PU × PCA) and the scenario-specific paths (e.g., Path 3: PT × ∼DS × PCA × DP)–are stable and reliable. The consistent results across different test settings eliminate the possibility that the configuration paths are caused by accidental data characteristics or subjective parameter settings, thus ensuring the validity of the study’s conclusions for explaining the combinatorial mechanisms of online healthcare community use.

**TABLE 7:** Online healthcare communities use factors that influence highly effective configuration outcomes.

Antecedent condition	UI					
	H1	H2	H3	H4	H5	H6
DS			⊗			
PCA		•	•	⊗		•
DT						⊗
DP			•		•	
PE	•	•			•	
PU	•	•		•		•
PR				⊗		⊗
IR				•	⊗	
PT	•		•	•		
Consistency	0.892	0.915	0.846	0.826	0.886	0.874
Original coverage	0.634	0.616	0.330	0.117	0.108	0.102
Uniform coverage	0.026	0.029	0.030	0.017	0.011	0.002
Global consistency	0.843					
Overall coverage	0.708					

“∙” means that the core condition is present; “⊗” means that the core condition is absent.

## 6 Discussion

From three aspects of patient factor, platform factor and doctor factor, based on qualitative comparative analysis research method, this study discussed the path configuration used by online healthcare community, and found 6 specific sufficient condition combination paths.

Among them, path 1 indicates that the perceived usefulness, perceived ease of use and platform trust of online healthcare community platform constitute the most important path for users to use online healthcare community. Path 2 indicates that the ease of use of online healthcare community platform, platform usefulness and patient evaluation are important paths for users to use online healthcare community. This shows that when users use the online healthcare community, the first consideration is the platform usefulness and platform ease of use, as well as the trust and patient evaluation of the platform (Wu et al., 2024; Zhang et al., 2014). For example, some scholars confirmed through regression analysis that perceived usefulness and platform trust are positively correlated with users’ online medical service adoption (Qin et al., 2024; Zhao et al., 2022), which is consistent with the core variables identified in our paths one and two. The research of this paper shows that in the environment of online healthcare community, platform perceived usefulness, perceived ease of use, platform trust, patient evaluation and other important paths for the same users to use online healthcare community.

Path 3 indicates that trust in online healthcare community platform, non-disease severity, patient evaluation and doctor professionalism constitute important paths for users to use online healthcare community. This indicates that trust in online healthcare community platform, patient evaluation and doctor professionalism are important paths for users to consider when using online healthcare community. Some scholars have shown that platform trust and platform evaluation are important factors influencing platform use (Nong and Ji, 2025; Yang et al., 2024; Zhang et al., 2023), but their study did not involve the regulatory role of disease severity. Our path 3 supplements this gap: when disease severity is low (∼DS), the combination of platform trust, patient evaluation and doctor professionalism shows higher explanatory power (original coverage 0.330), which is because Chinese users tend to use online platforms for minor illnesses or chronic disease follow-ups, and in this scenario, the synergy of doctor and platform factors becomes more critical–this is a nuance that single-variable regression research cannot capture.

Path 4 indicates that perceived usefulness, information richness, platform trust, non-perceived risk and non-patient evaluation constitute important paths for users to use online healthcare communities. Path 5 indicates that perceived ease of use, non-information richness and doctors’ professional quality constitute important paths for users to use online healthcare communities. Some scholars have shown that platform perceived ease of use and platform trust are important factors influencing platform use (Hiraga et al., 2025; Xin, 2025), but their study did not explore the substitution effect between variables. Our path 5 finds that when information richness is low (∼IR), perceived ease of use and doctor professional quality can still form an effective configuration (consistency 0.886), which means that for specific groups (e.g., elderly users with weak information processing ability), a user-friendly interface and trusted doctors can compensate for the lack of information richness–this “variable substitution” mechanism is difficult to identify in traditional linear models that assume additive effects of variables.

Path 6 indicates that perceived usefulness, patient evaluation, doctor trust and non-perceived risk constitute important paths for users to use online healthcare community. Some scholars have shown that platform perceived usefulness, platform evaluation and platform trust are important factors influencing platform use (Smith et al., 2025; Xu et al., 2022; Yang M. et al., 2022), but their research did not involve the impact of perceived risk. Our path 6 supplements this: the inclusion of “non-perceived risk” (∼PR) significantly improves the configuration’s consistency (0.874), which is because Chinese users are highly sensitive to medical information security and treatment risk, and only when risk concerns are alleviated can perceived usefulness and patient evaluation fully play their roles–this context-specific configuration further enriches the existing research on “perceived usefulness-risk” trade-off in online healthcare.

Among them, the most important combination of conditions is perceived usefulness × perceived ease of use × platform trust (original coverage: 0.634417; Consistency: 0.891789) because they present the highest raw coverage values. The results show that the realization of online healthcare community use is not driven by a single factor, but by patient factor, platform factor and doctor factor. The study found that no single factor could lead to the realization of the use of online healthcare community, but through the joint interaction of multiple antecedent conditions and the interaction of multiple factors, they jointly acted on the use of online healthcare community, thus explaining the inconsistent phenomenon of existing research conclusions to a certain extent [e.g., some studies found a significant effect of patient evaluation on usage, while others did not–this may be because patient evaluation only works in specific combinations, such as path 2 (PE × PU × PCA) or path 3 (PT × ∼DS × PCA × DP), rather than in isolation]. The configuration results show that perceived usefulness, perceived ease of use and platform trust (Krishna and Puram, 2025) play a core role in the process of influencing the use of online healthcare community, and promote the use behavior of patients in online healthcare community through synergistic interaction with other factors. This finding extends the TAM theory in the online healthcare context by revealing the “combinatorial extension” of core variables (perceived usefulness, perceived ease of use) with context-specific factors (platform trust, patient evaluation), rather than the single-variable extension in traditional TAM-based research.

## 7 Limitations and future research

This study focused on investigating the influencing factors and composing path of online healthcare community use based on FsQCA. Although outstanding contributions have been made in this study, there are also several limitations. Firstly, the study was constrained by the sample size and the diversity of participants. Although the sample used in this study was representative to a certain extent, it may not fully capture the nuances and variations in online healthcare community usage across different demographic groups and regions. Future research could expand the sample size and include more diverse participants to enhance the generalizability of the findings. Secondly, the study focused on a limited set of influencing factors and composing paths. While the factors and paths examined in this study are significant, there may be other unexplored factors or paths that could also influence online healthcare community use. Future research could delve deeper into other potential influencing factors and composing paths to gain a more comprehensive understanding of the phenomenon. Thirdly, this study excluded hospital-related factors and medical environment-related factors due to earlier challenges in measurement validity and factor independence, but this exclusion may have overlooked their actual impact on user usage willingness. Finally, this study focused on the current state of online healthcare community use, but the landscape of online healthcare communities is constantly evolving. Future research could explore how changes in technology, regulations, and user behaviors might impact the influencing factors and composing paths of online healthcare community use.

## Data Availability

The raw data supporting the conclusions of this article will be made available by the authors, without undue reservation.
